# Co-design Process of a Digital Return-to-Work Solution for People With Common Mental Disorders: Stakeholder Perception Study

**DOI:** 10.2196/39422

**Published:** 2023-01-18

**Authors:** Patrik Engdahl, Petra Svedberg, Annika Lexén, Carina Tjörnstrand, Catharina Strid, Ulrika Bejerholm

**Affiliations:** 1 Department of Health Science Lund University Lund Sweden; 2 School of Health and Welfare Halmstad University Halmstad Sweden; 3 Department of Psychology Lund University Lund Sweden

**Keywords:** co-design, mental health, mobile health, return-to-work, supported employment

## Abstract

**Background:**

Service users and other stakeholders have had few opportunities to influence the design of their mental health and return-to-work services. Likewise, digital solutions often fail to align with stakeholders’ needs and preferences, negatively impacting their utility. mWorks is a co-design initiative to create a digital return-to-work solution for persons with common mental disorders that is acceptable and engaging for those receiving and delivering the intervention.

**Objective:**

This study aimed to describe stakeholder perceptions and the involvement of a design process during the prototype development of mWorks.

**Methods:**

A co-design approach was used during the iterative development of mWorks. Overall, 86 stakeholders were recruited using a combination of purposeful and convenience sampling. Five stakeholder groups represented service users with experience of sick leave and common mental disorders (n=25), return-to-work professionals (n=19), employers (n=1), digital design and system developers (n=4), and members of the public (n=37). Multiple data sources were gathered using 7 iterations, from March 2018 to November 2020. The rich material was organized and analyzed using content analysis to generate themes and categories that represented this study’s findings.

**Results:**

The themes revealed the importance of mWorks in empowering service users with a personal digital support solution that engages them back in work. The categories highlighted that mWorks needs to be a self-management tool that enables service users to self-manage as a supplement to traditional return-to-work services. It was also important that content features helped to reshape a positive self-narrative, with a focus on service users’ strengths and resources to break the downward spiral of ill health during sick leave. Additional crucial features included helping service users mobilize their own strategies to cope with thoughts and feelings and formulate goals and a plan for their work return. Once testing of the alpha and beta prototypes began, user engagement became the main focus for greater usability. It is critical to facilitate the comprehension and purpose of mWorks, offer clear guidance, and enhance motivational and goal-setting strategies.

**Conclusions:**

Stakeholders’ experience-based knowledge asserted that mWorks needs to empower service users by providing them with a personal support tool. To enhance return-to-work prospects, users must be engaged in a meaningful manner while focusing on their strengths and resources.

## Introduction

The digital age has sparked a new hope that health care service users can be more involved and empowered in the delivery of their health care services [1]. Digital activities have become more common in primary health care and mental health services [2]. Drawing knowledge from the experiences of living with mental health problems is vital if digital initiatives are to be useful for users [3]. Leveraging users’ ingenuity through design has gained momentum in research and has proven to be effective in promoting well-being and alleviating illness, burdens, and health care costs [4,5]. User involvement in the co-design of health care services can generate numerous benefits such as improvement in patient choices, self-care, and positive effects on service delivery and patient outcomes [6]. Health care design planning is criticized for promoting exclusivity, and service users are not traditionally involved [4,7-10]. This is particularly conspicuous in mental health services [3]. Return-to-work (RTW) research has also been slow to adopt co-design principles in developing interventions, and accelerated adoption is expected to result in benefits. Most digital mental health solutions have not been researched or co-designed with users or other stakeholders [3,11]. Meeting the requirements of the stakeholders who deliver and use digital solutions is critical.

Common mental disorders (CMD) are one of the most frequent reasons for reduced work performance, sick leave, and increased risk of extended sick leave periods [12]. Depression and anxiety are the most commonly referred to [13], and sick leave is often recommended as a remedy [14]. Effective interventions that facilitate RTW are scarce, and promising interventions may be difficult to implement. This gap in practice has been shown to generate unnecessary struggle for persons with CMD regarding decreased mental health, empowerment, hope, and belief in the future [15-17]. However, converting RTW interventions into digital solutions holds the promise of providing person-centered support. It could reduce existing implementation barriers related to the structural complexity of welfare services and their lack of coherent support to users during the entire RTW process [18,19]. One such digital initiative is mWorks, intended to increase empowerment during the RTW process for people with CMD [18]. mWorks builds on a strength-based perspective and the individual enabling and support (IES) model for persons with CMD [20-23]. The IES model builds on supported employment (ie, evidence-based RTW intervention) as well as cognitive behavioral therapy (CBT) features, and is delivered by RTW professionals within health care systems. The IES model has been shown to be effective for RTW [24], empowerment, and depression [17], and users report that it applies a holistic and person-centered approach that provides hope and power during the RTW process [25]. Although supported employment has not been translated into a digital solution, CBT has been accessible as internet-delivered CBT over the past 2 decades. Guided internet-delivered CBT is equivalent to face-to-face CBT in reducing depression [26-29]. Furthermore, different self-management psychological interventions delivered through digital solutions have been shown to alleviate depression and anxiety symptoms [30-32]. Unfortunately, most digital solutions have not been co-designed with stakeholders and have failed to satisfy their requirements [11]. Therefore, available digital solutions for mental health services vary in user acceptability, engagement, and adherence [33]. Thus, entering a collaborative enterprise with stakeholders is crucial to meet the needs and values of service users [18,19,34].

The traditional RTW model entails various welfare services, eg, the social insurance agencies, public employment services, and workplace [35-37]. However, it tends to focus on diagnosis, functional disability, and activity limitations related to the sick leave certificate, which tend to generate a passive and weakening role with prolonged sick leave as a result [37,38]. This RTW model provides few opportunities to empower and tailor interventions according to individual needs and resources. mWorks encompasses a transition of the RTW approach from being fragmented to coherent, and from seeing patients as passive recipients to becoming valuable actors in their RTW process. It has the potential to fill the service gap by supplementing traditional RTW by providing service users with a digital tool to empower them during the RTW process [18,19].

To make mWorks useful in practice, developing a digital solution *with* (rather than *for*) users with a CMD is important [39]. Service users have traditionally not been offered the opportunity to influence the production of health care [4,7,9]. Consequently, research outlining the co-design process of digital solutions in the mental health field is scarce [3]. Even if different concepts, theories, and approaches that describe the coproduction process have emerged [7], researchers still struggle to implement coproduction activities in their research practice, which range from consultation, engagement, and participation in partnership, coproduction, and shared decision-making [10,40]. Therefore, co-design activities are warranted where users influence decision-making processes in mental health research to avoid tokenism and create digital solutions that have utility [3]. Thus, we applied a co-designed strategy and activities involving various stakeholder groups in the development of the mWorks. This study aimed to describe stakeholder perceptions and the involvement of a co-design process during the prototype development of mWorks.

## Methods

### Design

A co-design approach [40] was used to translate the IES model during the development of the mWorks. Co-design in research is based on a core philosophy of human rights involvement, democracy, equality [41], and the value of participatory efforts between researchers and stakeholders, with the goal of developing interventions that empower service users. Co-design also intends to identify a diverse range of needs and preferences, and lay the groundwork for successful implementation [1,4]. In this study, stakeholder participants acted as informants, co-designers, and evaluators, and provided their perspectives and feedback during the research process. The mWorks prototype development includes 7 iterative cycles of stakeholder involvement. These iterations included the initial preprototype, paper prototype, alpha prototype, and beta prototype. The study was conducted between March 2018 and November 2020.

### Research Context

This study was part of the research project *Work support in your pocket: Development and evaluation of mWorks, a digital mental-health intervention for return-to-work* [20] designed for persons on sick leave because of CMD. The project began with 2 formative studies that investigated stakeholder needs and preferences [18] and service user acceptability of a digital RTW solution [19]. This co-design study helped inform the development of mWorks, and the next step will be a testing phase where feasibility will be investigated with established process evaluation methods [42,43] before proceeding to a randomized controlled study design [44]. This research process conforms to the Medical Research Council framework for developing, testing, evaluating, and implementing complex interventions [35].

### Stakeholder Group Participants

Overall, 86 stakeholders participated in the iterative co-design process. Five stakeholder groups were involved: (1) service users (n=25, 29%) with experience of sick leave and CMD (eg, depression, including depressive episodes inherent in bipolar disorder and anxiety disorders). Three also had experience as professionals with digital system development (eg, software and gaming designers, app development in the private sector); (2) RTW professionals (n=19, 22%) representing CBT psychologists, occupational therapists or rehabilitation coordinators, and supportive employment specialists; (3) employers (n=1, 1%); (4) digital design and system developers at the regional or national level of the health care (n=4, 5%); and (5) public involvement (n=37, 43%) consisting of young adults (n=22) from a secondary school class who are frequent users of digital tools, and clubhouse members and mentors from the “Fontänhuset” (n=15), an organization with supportive employment as part of their social program. The age range for the whole group of participants was 18 to 65 years, with a mean age of 37 years and a gender distribution of 46% men and 56% women. All the participants had adequate Swedish literacy skills.

### Recruitment, Procedure, and Data Collection

#### Overview

Table 1 presents the purposes, stakeholders involved, and data collection types used in the iteration steps. The recruitment and study procedures are described as follows: Iteration 1 regarded the preprototype development phase and was administered as a workshop where national, regional, and local stakeholder groups took part. *Iteration 2* involved 4 cycles with 2 reference groups that elicited content features, function ideas, and materials that a user group iterated between each cycle. This rich material condenses during iteration 3. It was a workshop with various stakeholders who helped to discern the key content features and functions eligible for the paper prototype. The paper version illustrates central content ideas and a flowchart of mWorks that could be presented to software developers. In iteration 4*,* a downsized reference group tested the alpha prototype to provide feedback on the required refinement. During iteration 5, a secondary school class was invited to address content features and formats to elicit younger adults’ perspectives essential for ensuring mWorks acceptance for a broader age spectrum. In addition, clubhouse members and mentors helped to inform and broaden the understanding of usability and the needed refinements. Next, mWorks evolved into a beta prototype that, in iteration 6, was developed and refined through think-aloud interviews with service users. In iteration 7, the downsized reference group provided the final feedback. Researchers (UB, PE, and AL) were further involved in the strategic management of the County Council of Region Skåne, LU Innovation (ie, Lund University Innovation Department). The software development of the beta prototype was finalized through LU Innovation.

**Table 1:** Overview of data-collection procedures and iterations performed during the mWorks co-designed prototype development.

	Iteration 1: preprototype	Iteration 2: preprototype	Iteration 3: paper prototype	Iteration 4: alpha prototype	Iteration 5: alpha prototype	Iteration 6: beta prototype	Iteration 7: beta prototype
Data collection methods	Workshop with stakeholders	Reference and user groups	Downsizing workshop	Downsized reference group	Public involvement of young adults and clubhouse members or mentors	Think-out loud interviews	Downsized reference group
Purpose	To inform the preprototype draft in relation to legitimacy, relevance, and content features	To inform the development of content and features in relation to typical RTW^a^ frustrations (4 cycles)	To inform decision-making to identify and prioritize the most critical content features, format and functions needed to condense the material into a paper prototype, to support software development	To test the alpha prototype to inform what needed to be refined during software development	To inform the alpha prototype in relation to public terms of content features and format, and clubhouse members regarding acceptability	To inform usability dilemmas during software development of the beta prototype (users verbalized thoughts while using mWorks).	To test the beta prototype during software development to refine content and optimize function and usability
Participants’ recruitment	Stakeholder groups (n=11): 1. User, n=3 2. RTW, n=4 4. Digital, n=4	Stakeholder groups (n=16): 1. User, n=10 2. RTW, n=5 3. Employment, n=1	Stakeholder groups (n=6): 1. User, n=2 2. RTW, n=4	Stakeholder groups (n=5): 1. User, n=2 2. RTW, n=3	Stakeholder groups (n=37): 5. Public, n=37	Stakeholder groups (n=6): 1. User, n=6	Stakeholder groups (n=5): 1. User, n=2 2. RTW, n=3
Types of data	Audio recordings; field notes; mock-ups; photographs	Audio and video recordings; photographs; scribblings; field notes (4 sessions)	Audio recordings; photographs; scribblings; field notes	Feedback sheets (Word document), comments and bullet points together with screenshots of digital content and features	Audio and video recordings; photographs of mock-ups; field notes	Audio and video recordings; fieldnotes; open-ended interview questions, eg, did you understand how to use mWorks? Was something easier or more difficult to do? What would you change?	Feedback sheets (Word document) with bullet points and related screenshots of digital content and features

^a^RTW: return-to-work.

#### Recruitment

Purposeful sampling was used to recruit participants throughout the development process [45]. Initial contact was initiated via email with previously known stakeholders. The exception was public involvement (iteration 5), where convenience sampling was applied because of stakeholder availability. A secondary school teacher and clubhouse personnel were contacted by email and informed of the study. In turn, personnel informed researchers that stakeholders agreed to participate and suggested a time and date for the meeting. During iteration 6, a total of 4 service users from previous formative research [18,19] participated in the think-aloud interview [46]; 3 were represented in the user group. In addition, 1 service user was recruited via a flyer published in the general campus area (Lund University), and 1 via a nonprofit user organization, where personnel asked potential participants about their interest in participation. They were then contacted via phone or email. Among the invited stakeholders, only 1 employer and 1 service user declined participation.

#### Pedagogic Materials

The pedagogical material involved a preprototype with initial design ideas and typical frustrations in the RTW process that were used during iterations 1 and 2. The preprototype pedagogic material was produced by UB and involved initial design ideas synthesized from earlier IES research [24], mapping out typical frustrations of the RTW process as reflected by individual interviews (n=60) of persons with CMD, previously known factors for the RTW process [25], and formative research on stakeholder preferences, needs, and acceptability of mWorks [18,19]. Identified frustrations with the RTW process were: (1) “I see no way forward,” (2) “I don’t believe in myself and have no energy,” (3) “There is no support available,” (4) “Talking about my mental health is difficult,” (5) “I do not know where to start,” (6) “I do not have any strategies that work,” and (7) “I keep feeling bad at work (when I returned to work).” In addition, 2 personas (Max and Sara) were developed to represent service users in an archetypical way and thus help stakeholders incorporate diversity regarding demographic factors, diagnoses, gender identity, delivery context, job type, psychosocial workplace environment, and typical frustrations [47]. They were used to personalize the service users’ needs and preferences in iterations 1-5 and 7.

#### Iteration Procedures

Between iterations, the data synthesis process was led by UB and iterated among the authors (PE, AL, and CT). This data collection procedure allowed us to feed forward information during the refinement of the prototype development.

#### Preprototype

The co-design procedures began with a preparatory meeting with the national organization (Inera) responsible for the coordination and support of the digital business development of municipalities and regions in Sweden. Iteration 1 introduced stakeholders to the research project, preprototype, initial design ideas, personas, and identified typical frustrations through the PowerPoint presentation. Stakeholders were then divided into 2 representative groups and provided with sticky notes, differently colored pens, large writing papers, and Max and Sara posters. They were prompted to discuss an open-ended question about frustration with the RTW process and what features were needed to support users’ return to work. Different features of mWorks were highlighted separately (ie, design, content, usability, and potential needs of the service user) to facilitate discussion, and sticky notes were used to prompt, stimulate, and generate ideas. The workshop sessions lasted 90 minutes.

Iteration 2 was performed using a reference group and user group. This co-design phase involves 4 subsequent cycles, each representing 2 of the 7 typical frustrations. The reference group acted as a design partner, whereas the user group provided feedback on PowerPoint materials that were developed between each workshop. A requirement list was created between sessions to merge the preprototype and evolving co-design ideas. The cycles allowed us to oscillate between ideation (reference group) and validation (user group) to inform the paper prototype version so that it matched the stakeholders’ needs, values, and requirements. The reference group met at the beginning and end of each workshop to be introduced and to conclude the main co-design ideas. During the main part of the workshop, the group was split into 2 groups that represented all stakeholders. They were encouraged to discuss frustrations and their solutions through redemption scenarios (Multimedia Appendix 1) [48,49]. For each cycle, the reference group sessions lasted 120 minutes and the user group sessions lasted 60 minutes.

#### Paper Prototype

The 90-minute downsizing workshop with stakeholders, iteration 3, was critical for the decision-making process and determining what in the list was critical for the IES model and RTW process (Table 1). The session started with a PowerPoint presentation of the merger of the preprototype and co-design materials from iterations 1 and 2*,* and again Max and Sara were highlighted. The workshop was audio-recorded, and stakeholders’ scribblings and drawings were photographed. The final paper prototype PowerPoint presentation was then shown to LU Innovation and the software company that developed the alpha prototype.

#### Alpha Prototype

For iteration 4, a downsized reference group of stakeholders was consulted to provide feedback on mWorks usability and content features, formats, and functions during the programming process (Table 1). Stakeholders were provided access to the alpha prototype and asked to complete specific tasks, eg, to provide written comments and bullet points on usability aspects of effectiveness (ability to complete a task in a specified context), efficiency (ability to complete a task with accuracy), and satisfaction (perceived comfort and pleasantness during interaction) [50]. The written comments are summarized next to the related screenshots of digital content and features. Each response took approximately 45 minutes.

Involving the public was the main purpose of iteration 5 and comprised workshops with young adults from a secondary school class, as well as members and mentors of a clubhouse who were familiar with RTW. The students were divided into 3 groups and given tabletop devices to interact with the alpha prototype. Each group discussed topics such as esthetic design, content features, formats and functions, motivation to use the prototype, and navigation. They were prompted to place post-it notes on their mock-ups. The workshop took 90 minutes to complete. The clubhouse workshop was led by a service user and a researcher who began with a PowerPoint presentation about the project, project development, and the alpha prototype. Members and mentors who were familiar with the RTW process were prompted to determine the critical implementation factors for real-world usage.

#### Beta Prototype

Iteration 6 included think-aloud interviews with 6 service users with experiences of CMD and sick leave. Users were evenly distributed among the 3 subgroups that tested different sections of mWorks. They were asked to perform various predefined tasks and verbalize their thoughts, feelings, and experiences during use according to the think-aloud method [46]. During the think-aloud session, each user was asked a series of open-ended questions about their experiences accompanied by probing questions. Approximately 85% of all usability errors were found among 5 to 6 participants [51].

For the last step, iteration 7, the same downsized reference group, and procedure from *iteration 4* were used, focusing on the final refinements of alpha prototype programming. The researchers again compiled and processed comments to allow for refinement before testing the prototype in a real-life context.

### Data Analysis

All data were analyzed using qualitative content analysis, inspired by Graneheim and Lundman [52]. This analysis method is preferable for capturing a variety of data sources and patterns when dealing with a large data corpus [53]. The initial analysis process involved listening to and watching recordings, studying visual data (ie, photographs, scribblings, and mock-ups), and reading the field notes to gain a comprehensive understanding of the data. Next, the recordings were transcribed verbatim and visual data were observed and described in words for merging into sections with other text portions. This data treatment was performed by PE, which made it possible to extract units of meaning associated with delivery and content features, functions, and formats that were ascertained during the coproduction process. These meaning units were subsequently condensed, but still represented the original statement. The condensed meaning units were then coded and assembled into subcategories as assessed by PE, UB, and PS. The subcategories were then grouped into the main categories and assigned to themes to allow for higher degrees of abstraction and interpretation. For example, 1 stakeholder explained the need to clarify the purpose of the mWorks content concerning RTW. This was coded as *Clarifying mWorks purpose for RTW*. Codes representing need and meaning were assembled into the subcategory *Explaining the significance of mWorks content concerning RTW*. These subcategories were subsequently grouped into the category “Facilitating comprehension of mWorks.” Finally, categories with similar topics were assigned the theme *Improving service user engagement.* Categories and themes were processed until a consensus was achieved among all authors.

### Ethical Considerations

Before participating, the study participants received oral and written information about the overarching research project and implications of their involvement in this study. Before data collection, each participant was informed of the purpose of the current iteration session and provided informed written consent in accordance with the ethical principles of the Helsinki Declaration of 1975, as revised in 2000 [54]. Ethical research approval for the mWorks project and this study was obtained from the Regional Ethics Committee of Lund, Sweden (Dnr 2017/324).

## Results

### Overview

The stakeholders’ experience-based knowledge and perceptions gained from the co-design process resulted in 3 themes: (1) empowering the service user back to work, (2) providing service users with their own personal support tools, and (3) improving service users’ engagement. These themes informed the prototype development of mWorks (Table 2).

**Table 2:** Themes, categories, and subcategories from 5 stakeholder groups during the co-design process of mWorks.

Theme	Category and subcategory
Empowering the service user back to work	Enabling self-management back to work Supplementing traditional RTWa and health care services Providing a comprehensible overview of the RTW process Coordinating the support network to facilitate RTW Resolving ambivalence regarding mental health disclosure Fostering service user control Breaking the downward spiral Assisting device that identifies strengths and resources Helping to reshape a positive self-narrative Permeating a positive, hopeful, and stigma-free impression Perceiving the encounter as warm and welcoming
Providing service users with own personal support tool	Mobilizing own strategies Coping with thoughts and feelings during work return Helping to identify cognitive strategies Suggesting a variety of content features Helping users to plan for their RTW process Improving data privacy Implementing measures to safeguard personal data Requesting options to interact with self-selected support persons
Improving service user engagement	Facilitating comprehension of mWorks Need to understand content intuitively Explaining the significance of mWorks content in relation to RTW Reducing the amount of text-based content Reducing the need for recall Need for accessible chat support Providing motivation and goal-setting strategies Addressing service users’ jaded motivations Presenting a time-bound, measurable, and concrete development process Importance of a goal and reward-oriented design Advising for a more engaging design

^a^RTW: return-to-work.

The five *stakeholder groups* included: (1) service users, (2) RTW professionals, (3) employers, (4) digital design and system developers, and (5) public involvement.

### Empowering the Service User Back to Work

#### Overview

One of the most critical design choices during preprototype development (Table 1) was to consider the role of mWorks as the service user’s own self-management solution. Because of legal barriers to information exchange between service users and RTW actors on the same digital platform, self-management has been identified in a related national project by one of the included national stakeholders [55]. Consequently, mWorks needs to be solely a self-management tool focused on strengthening and empowering the individual, centered on the service user’s capabilities, providing accessible information, and supporting engagement in the RTW process. In addition, a secondary requirement was that the mWorks design should meet the needs of a broad audience to enhance adoption. Therefore, we iteratively tweaked the color scheme of the interface to be more esthetically pleasing.

#### Enabling Self-management Back to Work

*Supplementing traditional RTW and health care services* by providing a self-management tool with accessible information and support was crucial for mWorks from the initial preprototype development, *iteration 1* (Table 1). By filling the service gap with personalized RTW support, mWorks was anticipated to enable service users to self-manage by strengthening and empowering them. During later iterations, one stakeholder validated that mWorks could supplement traditional RTW services and assist them in their daily work:

(mWorks) It is a complement to health care. I think you should sell this to all outpatient clinics because it can be a very good aid for us.

Iteration 6, service user

The importance of *providing a comprehensive overview of the RTW process* was emphasized during the development of the paper prototype (Table 1). Stakeholders explained that an overview could serve the purpose of (1) aiding understanding and use of mWorks and (2) providing information about the RTW process, including key events important for the service user. They suggested that overview information be delivered by a professional who delivers mWorks or in written format as an integral or external (ie, written manual) part of mWorks. They advocated mWorks to cover all vital steps of the RTW process instead of single aspects. Providing comprehensive information was anticipated to provide a safe space for service users to think and strategize subsequent RTW steps. During the think-aloud interviews, one stakeholder validated this idea and emphasized the advantages of including everything related to RTW in one comprehensible digital solution:

What I have seen seems very good. It includes all the essentials and leaves room for some special adaptation.

Iteration 6, service user

During the development of the paper prototype, *coordinating the support network to facilitate RTW* was commonly considered vital (Table 1). Reference group stakeholders (*iteration 2*) remarked that service users sometimes experience frustration with the uncertainty of not knowing what and from whom they can receive RTW support. They suggested content features to help mind-map crucial RTW persons, for example, as professionals or related parties of their social network, who understood the service user’s unique needs and preferences. By aiding in the identification of crucial persons, users could recognize and define the support they could provide.

To define the network. Whom do I have around me, and what is everyone’s function?

Iteration 2, service user

By allowing service users to comment when an interaction takes place, the content feature could also aid service users in evaluating the adequacy of support. Ideally, an interactive communication function is necessary to allow service users to invite different RTW professionals to communicate with them. However, because of legal restrictions (highlighted in iteration 1), it seemed adequate for service users to assemble their networks. It would give them a better understanding and overview of who and what these related parties or professionals could assist with and share with these individuals.

*Resolving ambivalence regarding mental health disclosure* was discussed during the preprototype development, and it was thought necessary to feel in control of the RTW process. This feature was anticipated to facilitate managerial and collegial support, allowing colleagues and managers to increase their mental health literacy and understanding of service users’ workplace support needs. Thus, mWorks could be used preventively and as an early intervention if their concerns were verbalized and communicated early. During preprototype development (*iteration 2*), stakeholders validated the need to provide this disclosure decision-making tool so that users could reflect and document to whom, what, and how to share their mental health information. For example, identifying which colleagues they could confide in was perceived as a critical feature of mWorks:

It is important to receive decision-support about whether to talk (about my own mental health) by hearing the stories of others but also asking questions that might prepare me. This way I can obtain an idea of what they (managers and colleagues) are interested in knowing.

Iteration 2, employer

Positive examples in a video format (infographics) of what others found helpful were preferable in answering these questions and providing adequate solutions. These examples were presented as success stories of peers discussing their mental health. This would inform users of the pros and cons of self-disclosure, and what doing so may entail. However, the user group had mixed thoughts about the need for videos that provided education about depression and anxiety, because they were perceived as burdensome to view alone and without a trained therapist:

It is crucial that the support not only illustrates the risks but also shows the positive impact on others in some narrative format. That this is built into the app—what others have perceived was preferable to tell. It is essential that the app can provide what others thought was positive to tell.

Iteration 2, psychologist

*Fostering service user control* over the use of and approach to mWorks was generally viewed as positive and something to aspire to throughout the development process. Although some individuals preferred to be tutored in the use of mWorks, others were in favor of using it independently. Therefore, it seemed reasonable for users to choose whether and to what extent they received human guidance, or how much they wanted to control themselves. Some stakeholders thought that the required steps and forcing users in certain directions might be associated with external control and result in a loss of retention. Others have emphasized the need for an open design with a clear framework and guiding steps to maintain control. One stakeholder in the user group (iteration 2) opposed the idea of being able to use all features at once. They feared that this would be overwhelming, difficult to understand, and result in immediate cessation of use. Gating content step-by-step and eventually unlocking more content were suggested as the user progressed during RTW.

#### Breaking the Downward Spiral

One commonly discussed frustration during RTW was the absence of belief and confidence that service users have about their ability to return to work (ie, self-efficacy). Turning these negative thoughts into a positive sense of self and learning about their situations and resources were considered critical steps in facilitating RTW. Therefore, mWorks must be an *assisting device that identifies strengths and resources* to help service users to understand their capabilities. Asking users to list their past and present strengths and resources was an appreciated idea. These strengths and resources could be documented in dialogue with RTW professionals and employers, for example, as curriculum vitae, work profiles, and work abilities. Stakeholders in the reference group (*iteration 2*) proposed that mWorks regularly ask questions to help service users identify their resources:

If mWorks have different questions you answer, you can have a list of all the resources you have answered for the past three weeks. “What have you done today that worked?”, “I baked a cake.” Yes, then organization and planning; the application sums that up for you. “I'm good at planning.”

Iteration 2, RTW professional

Identifying strengths and resources would effectively enrich dialogue with the employer, be used to identify workplace strategies, and match work tasks. The user group (*iteration 2)* validated this suggestion but said that it might be challenging to identify strengths if you felt down and self-critical. Therefore, RTW professionals, employers, and related parties can help users identify their strengths and jointly build a work profile.

*Helping to reshape a positive self-narrative* is vital for enabling the RTW process. It was thought that changing one’s self-view would prompt service users to address their motivation to RTW, and the goals this would entail. Motivational, cognitive, and behavioral strategies to prepare for change and goal-setting, which are also present in the IES model, were recognized as important initial tasks during pre- and paper prototype development (Table 1). Asking service users: (1) What has happened in relation to the workplace and sick leave in the past? (2) Where am I now? (3) How and where am I going when returning to work? These questions would help to structure a more accurate self-narrative, motivate the user, and help them attribute meaning to RTW. Peer success stories can help them identify the steps in their journey. These narratives might provide service users with a positive identity, sense of normality, understanding of their mental health, and hope for the future.

Stakeholders in the reference group (*iteration 2*) validated service users’ frustration with the everyday experience of lack of hope for the future and that they “see no way forward” during the sick leave period. Many agree that service users typically have a negative outlook on the RTW process and experience a lack of hope for the future. Thus, it was described as crucial for *permeating a positive, hopeful, and stigma free impression* when interacting with mWorks**.** Stakeholders explained that a strength-based and empowering approach to user needs could be accomplished by RTW professionals delivering mWorks, but present in mWorks’ software content and design.

Throughout development and most often during pre- and alpha prototype development (*iterations 2 and 5)*, stakeholders agreed on the importance of *perceiving the encounter as warm and welcoming* if users were to stay engaged with mWorks. They described how small things such as mWorks greeting the users by saying “Hello ‘Name,’” or having a pop-up window as how the day had been could make an important difference. The experience of understanding, seeing, and taking it seriously is essential. Stakeholders believed that service users’ negative reflections could be alleviated by promoting positivity through value-based languages. During the alpha prototype development, young adults reported that instilling hope, warmth, and empowerment were essential ingredients in promoting a positive mindset:

It (mWorks) must be positive when you enter the app. So, that it is not something I cannot follow through, and I click through it quickly without it being useful.

Iteration 5, service user

### Providing Service Users With Own Personal Support Tool

During the initial preprototype development and later the alpha prototype, it became apparent that mWorks should focus on an individual’s personal needs and preferences. Helping service users manage their mental health, identifying and adopting cognitive strategies, coping with difficult thoughts and feelings, and planning for eventual work return were developed as crucial features of mWorks.

#### Mobilizing Own Strategies

During preprototype development (*iterations 1 and 2*), stakeholders agreed that mWorks should help service users find useful strategies to *cope with thoughts and feelings during work return* and when at work. mWorks can be used to help identify and map stressful emotions concerning past and present experiences and understand their triggers. In addition, stressful emotions must be linked to ways of thinking and solutions with specific strategies. For example, one stakeholder suggested to “remind the user to take a walk or do physical activity before the workday to feel calm and more positive.” In contrast, a stakeholder from the service user group rejected a feature that mapped stressful situations. They stated that confronting one’s feelings and emotions could be frightening and overwhelming.

The reference group members (*iteration 2*) believed that a central task of mWorks should be *helping to identify cognitive strategies* attuned to user needs and preferences. They explained that service users should have access to a toolbox to deal with the difficult thoughts and emotions associated with their mental health. For example, CBT strategies could be used to cope with negative thoughts and feelings or adjust avoidant behaviors. mWorks could prompt users to ask friends, relatives, or coworkers about good strategies. The user group agreed with the necessity to access adequate strategies but stated that they should be individual and voluntary to be shared with whomever they prefer:

CBT accompanies me to work, and I do not have to share it to my employer. Sharing (information) with employers should be voluntary.

Iteration 2, service users

Strategy storage and reminders are recognized as essential features. Reminders could improve adherence to exercises and help maintain behaviors and habits that serve the user in their RTW process and mental health. During preprototype development (*iterations 1 and 2), the stakeholders suggested various content features* that support the RTW process, which could be individualized according to needs and preferences (eg, adjusting the order for content completion). For these reasons, mWorks was developed to use a person-centered approach:

The possibility of adapting the modules individually, because that may not be the case. As an example, I might not want to work on my anxiety right now, but instead I may want to work on something else. That there is a smorgasbord in some way that I can choose from, to work with these parts because they are important to me right now. This is also a motivating factor.

Iteration 1, psychologist

Among the plethora of suggested content features was a “first aid” button. This button functions as an early intervention when service users experience stressful situations. They could use the function of voice control when experiencing fatigue or when preferring to talk (rather than write). Stakeholders wanted external links to useful tools that could supplement mWorks’ content. This idea was not incorporated because stakeholders in the service users group advocated for mWorks to be easy to use, learn, and understand. They feared that links to external applications would sidetrack users from achieving that goal.

During the think-aloud interview (*iteration 6*), stakeholders endorsed that mWorks should provide an overview of the progress of service users’ journey back to work. They considered this an important feature for improving the RTW prospects. Stakeholders explained the importance of providing a clear structure by *helping users to plan for their RTW process*. This was consistent with previous solutions suggested during preprototype development (*iteration 2*). mWorks could help service users plan, prioritize, and manage their time via a to-do list and schedule, thereby helping them structure their everyday lives. The scheduling time for recreation between activities related to RTW was equally important. It was suggested that mWorks reward small steps and goals toward the RTW:

Wouldn’t it be a great feature to have a scheduler that helps you specify your time (management)? If you have not planned a break, it is a warning sign that you are quite stressed and have been stuck in the to-do list swamp, which is only suffering.

Iteration 2, psychologist

#### Improving Data Privacy

Stakeholders frequently pointed out that all registered information in mWorks must be securely stored and have secure login features, such as a two-step verification process (*iteration 5*). Thus, *implementing safety measures to safeguard personal* data is important, because mental health information is considered sensitive. Except for the user, no one should have access to their information in the mWorks. Assuring data privacy maximizes servicer user trust and adoption. Although service users prioritized the safety of their data, options to *interact with self-selected support persons* were desired. To share data via mail, chat, or forum formats to discuss dilemmas and procure support from a select group of people (such as mentors, peers, or professional therapists).

### Improving Service User Engagement

#### Overview

During the later iterations, it was apparent that facilitating an understanding of the mWorks content and its purpose concerning RTW was necessary for service user engagement. To reduce cognitive demand, a chatbot was designed to deliver the content in bite sizes and explain why completing certain features could advance the RTW process. In addition, the design of mWorks must meet the requirements of a broad audience to improve its adoption. We also iteratively tweaked the interface color scheme to make it more esthetically pleasing.

#### Facilitating Comprehension of mWorks

During *iterations 4 to 5*, the mWorks alpha prototype was viewed as easy to learn and maneuver. Even so, stakeholders explained the importance of *needing to intuitively understand mWorks content*. When stakeholders logged into mWorks for the first time, they had trouble grasping the “five steps to work.” As a result, the app’s purpose was vague and unclear to the user. Implementing a tutorial that explained the overarching content and goal was suggested to set the stage, provide a quick guide to use and navigate, and address the meaning and motivation for progressing back to work. Superfluous graphical content was removed to clarify the first landing page, as suggested during the public involvement:

When you start the app, can’t you then have a quick overview that appears in a small bubble at each step? This is how it should be: here you will find your resources, here comes your network, and here you can view a person in the same situation as you. Then, you get a small summary that must look sickly nice. Otherwise, you will just zip past it.

Iteration 5, service user

Likewise, *explaining the significance of mWorks content in relation to RTW* was considered important for user engagement. Stakeholders in the think-aloud interview (*iteration 6*) asked themselves *why* the content was meaningful for RTW, eg, why reflecting on the lived experience of previous and current work situations could increase RTW prospects. An unclear direction contributed to meaninglessness toward task completion and the prioritization of content features. One stakeholder commented:

There is no clear idea about (the) purpose. Why should I use this (mWorks), and what can it provide for me? Some info about the different steps (would be useful). Here it only says what it helps with—“this leads to what you can (do).” If we take My Resources for example, “it helps you to highlight your resources and formulate a work profile.” Why?

Iteration 6, service users

They further explained the importance of *reducing the amount of text-based content* during think-aloud interviews (*iteration 6*). During the limited time they had to familiarize themselves with mWorks, service users felt overwhelmed because they had to read and write frequently. They found this cumbersome and inhibited the user from learning and completing individual tasks in the mWorks. Therefore, service users requested that the information be presented in a “bite-sized manner” and have predefined answers:

It’s a lot of empty boxes. When you see that, you think, these are all things I need to do (sigh). Do I really need to fill in more? I find “My strengths” more appealing. There you can see examples in the boxes themselves.

Iteration 6, service users

During the think-aloud interviews (*iteration 6*), *reducing the need for recall* was considered important to lower the cognitive load. This was emphasized when users had to write something and simultaneously retain the information box and examples tied to the same feature. The reduced reference group agreed that there were many empty text boxes for the user to fill in the beta prototype, and worried that the workload would be too great and result in a loss of engagement:

The threshold and energy it takes to motivate myself is too great. Feel free to explore ways that make it easier for me to start filling in information.

Iteration 7, service users or game designer

*Needing accessible chat support* was important to avoid frustration related to use when bugs and errors emerged. Throughout the development process, stakeholders explained that people with depression and anxiety would stop using mWorks if support was not readily available.

#### Providing Motivation and Goal-Setting Strategies

During the development of the preprototype*, addressing service users’* *jaded motivation* was perceived as a prerequisite for facilitating RTW. To alleviate this lack of motivation, stakeholders agreed that asking simple questions about what motivates service users to return to work is a promising solution in accordance with motivational interviewing communication techniques. Once service users had started their RTW process, feedback through diagrams or visible forward steps needed to be included so that they could see that they were making progress and maintaining momentum:

Some sort of overview of what I have succeeded with (is needed)... That you fill in what you have actually done. Because sometimes you can feel that you haven’t done anything. But when you can look at it (mWorks), and see that I actually did this, this, and this, it may not be what I set out to do, but I did a lot anyway. So, it’s some kind of progress list.

Iteration 2, service user

Stakeholders in the preprototype development (*iterations 1 and 2)* explained the importance of *presenting a time-bound, measurable, and concrete development process*. Ideally, this would be presented step-by-step to condense larger goals into feasible subgoals while providing service users with a direction to move toward RTW. Otherwise, stakeholders feared that service user motivation would suffer because there was no visible end to their journey, contributing to a sense of meaninglessness.

Stakeholders were *prioritizing the importance of a goal and reward-oriented design* throughout the development process to provide opportunities for users to experience an increased sense of motivation. Condensing large steps into smaller substeps is proposed as more manageable. These small steps enabled service users to act and implement them. In addition, mWorks could reward service users to complete steps and provide a road map to illustrate their progress and trajectory toward RTW.

The stakeholders, considering young adults’ futures during alpha prototype development (*iteration 5)* explained conflicting ideas about how to design mWorks. Although some thought that the color scheme was bright and warm and produced a calm and positive feeling, others perceived the colors as numb and boring. The 5-steps-to-work took the shape of a flower during the alpha prototype and was regarded as childish and derogatory to a younger audience:

And not directly a flower. It’s too childish. If it had been a kid who goes into this app, they would have thought it was a game.

Iteration 5, service user

Young adults were *advised* *for a more engaging design* and requested options for choosing different color schemes and patterns. They believed that the challenge lies in finding a design that provides the same positive feelings without belittling users.

## Discussion

### Principal Findings

The involvement of stakeholders in the co-design process permitted the accumulation of vital experience-based knowledge for consideration during the development of the mWorks. The importance of providing a digital solution that strengthens service user empowerment and control during the entire RTW process was frequently raised during the iterations and the pre- and paper prototypes. This was identified as one of the main strengths of mWorks. These findings are in line with a new systematic review of digital mental health applications that highlights the benefit of improving the service user’s locus of control by developing self-management skills [33]. Similarly, a central finding indicates that mWorks may serve as supplementary support to traditional RTW services and enable self-management during the RTW process.

The provision of service users’ personal RTW tools was essential during development because of the nonlinear nature of the RTW process. This idea provided a rationale for designing mWorks with various features that cover the entire RTW process, available from the initial login session, to mobilize their own strategies back to work. Service users are nudged to work through the content features in a stepwise manner but are free to use mWorks according to their preferences, just as in the individual enabling and support model [24]. A potential drawback of this option is the risk of feeling cognitively overwhelmed, which could negatively impact service-user engagement [18,19]. Other studies have similarly remarked on the inherent tension between user autonomy and when clear guidance is needed to optimize service-user engagement [33,56].

When service users initiate the use of mWorks, it is crucial that they clearly understand the content and overall purpose to ensure their engagement. Data indicate that service users spend approximately 5 minutes trying to understand new digital solutions before discontinuation [57]. Thus, it is vital to facilitate the meaning-making process to ensure sufficient engagement and reduce attrition levels. Previous co-design efforts of digital solutions to improve mental health and well-being similarly argued for the benefit of providing a clear purpose to improve engagement [18,19,58]. This study further explained the necessity of specifying *why* a specific subtask is meaningful for the overall goal of RTW. On the basis of the findings, our solution to facilitate the comprehension of mWorks’ purpose was to provide subtasks with additional context via a chatbot. The literature suggests that artificial intelligence–directed conversational agents, that is chatbots, can enhance understanding and engagement levels and are promising automated alternatives to human support [58,59]. Further investigation needs to focus on whether these digital alternatives to human support provide improved engagement and positive outcomes, which have been promoted as particularly valuable for increasing the scalability of digital solutions [29].

### The Co-design Approach

Co-design efforts with genuine stakeholder involvement are anticipated to generate more acceptable and engaging interventions with greater utility for those receiving and delivering them [41]. We intended to develop mWorks *with* stakeholders, not *for* them [39]. However, even if different concepts, models, and theories of co-design generally corroborate that higher levels of stakeholder involvement are desirable [7,10,39,41], there remains ambiguity regarding how to achieve sufficient involvement during the entirety of a research project. This is especially true in this project, because the prototype development involved translating the IES model into a digital format rather than designing the prototype from scratch. Smits et al [10] suggest the importance of cementing the anticipated roles and expectations of stakeholders and researchers to form an authentic and sustainable partnership throughout the research process. As Nygren et al [40] explain, this emphasizes that stakeholders stay engaged over time to achieve an appropriate degree of involvement. Although desirable, it is seldom feasible for stakeholders to remain engaged during the entirety of a research project because such endeavors are usually long processes that take place over several years. A pragmatic solution would be to recognize the diverse forms of involvement that can manifest throughout the co-design process [10]. Relying on diverse and innovative forms of involvement beyond traditional methods has been identified as a prominent factor in overcoming involvement barriers in co-design research [7]. This study on mWorks is a good example, where the ingenuity of stakeholders was allowed to influence the intervention development during the research procedures [4,5].

### Methodological Considerations

Our work shows that it is possible to translate and co-design the development of a digital solution for work return based on evidence-based methods such as CBT and SE to promote service users’ influence over their RTW process. This study provides novel guidance for researchers seeking to coproduce digital solutions using stakeholders as co-design partners. However, there are some limitations that should be considered. We recruited stakeholders using nonprobability sampling methods throughout the study. This can be criticized for being less stringent than probability sampling methods [45]. However, the chosen sampling methods were the most time-efficient and allowed us to find participants with adequate subject expertise to generate valuable knowledge for each iteration, which could further inform the development of mWorks. A large sample size with a diverse range of stakeholders represented only one employer in the entire sample. Thus, insights from employers are limited, affecting our findings’ transferability [60], which is a subject for future research.

Stakeholders’ difficulties in comprehending mWorks during the iterative development process were partly a consequence of methodological limitations because they had a limited timeframe to familiarize themselves with and were only subject to specific parts of mWorks. Thus, it was difficult for them to immediately grasp the overall RTW process. In addition, the initial delivery procedure of mWorks to service users is planned to last for 10 weeks, with continued use for up to a year, and would thus benefit from stakeholders being present during the entire research procedure.

As mentioned in the *Methods* section, the research group met between each reference group workshop and compiled a requirement list. This may have contributed to biased interpretations. To remedy this threat to credibility, each researcher synthesized the data separately, compared the summary texts, and worked out the most important factors until a consensus was achieved. Subsequently, the requirement lists were presented to the reference group during an upcoming workshop to allow them to check our interpretations [60]. This allowed the research group to eliminate biased interpretations and strengthen the credibility of the findings. However, our co-design approach involves collaboration between researchers and stakeholders throughout the development process. This allowed for continuous iteration and refinement of mWorks to ensure that the content and design corresponded with service users’ needs and preferences.

### Conclusions

By leveraging the ingenuity of stakeholders, this co-design study provides direction on how and what to include in the prototype development process of a digital RTW solution for persons with CMD. Stakeholders’ experience-based knowledge was vital in informing mWorks development, and showed the need for an empowering digital solution that provides service users with their personal support tools and focuses on their strengths and resources while engaging them in a meaningful way to achieve RTW. This study and the co-design approach can inform researchers in future studies where stakeholders are partners in research.

## Appendix

Multimedia Appendix 1 Example of a redemption story worksheet used to extract stakeholder ideas and solutions to the return-to-work process's predetermined "no way forward" frustration.

## Abbreviations

CBT: cognitive behavioral therapy
CMD: common mental disorders
IES: individual enabling and support
RTW: return-to-work
