# Supplementary figures and images for: Co-design Process of a Digital Return-to-Work Solution for People With Common Mental Disorders: Stakeholder Perception Study
Source: JMIR Form Res. 2023 Jan 18;7:e39422. doi: 10.2196/39422 (PMC9892984; doi:10.2196/39422)

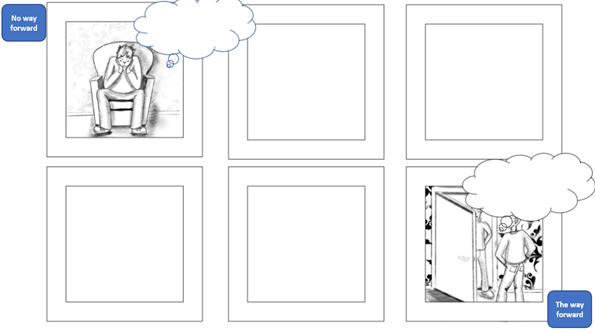

Supplement: Multimedia Appendix 1 [file formative_v7i1e39422_app1.png]
